# Pollen Feeding Reduces Predation of Northern Corn Rootworm Eggs (Coleoptera: Chrysomelidae, *Diabrotica barberi*) by a Soil-Dwelling Mite (Acari: Laelapidae: *Stratiolaelaps* *scimitus*)

**DOI:** 10.3390/insects12110979

**Published:** 2021-10-29

**Authors:** Deirdre A. Prischmann-Voldseth, Stephanie J. Swenson, Robert Brenner

**Affiliations:** 1 Department of Entomology 7650, North Dakota State University, Fargo, ND 58108-6050, USA; Stephanie.Swenson@uni-kassel.de (S.J.S.); Robert.brenner@wsu.edu (R.B.); 2 Department of Botany, Institute for Biology, University of Kassel, Heinrich-Platt-Strasse 40, 34132 Kassel, Germany; 3 Department of Entomology, Washington State University, P.O. Box 646382, Pullman, WA 99164-6382, USA

**Keywords:** sunflower, *Helianthus*, pollenivory, oophagy

## Abstract

**Simple Summary:**

Flowering plants are used to enhance pest control by predators and parasitoids, but insect herbivores can also use floral resources. We documented the species of adult rootworm beetles on key plants, and found that northern corn rootworm (NCR) adults were commonly associated with sunflower inflorescences while western corn rootworm adults were most abundant in corn and on squash blossoms. Consumption of sunflower and corn pollen by NCR adults did not impact predation of their eggs by an omnivorous mite, but a predatory soil-dwelling mite ate pest eggs less frequently and took longer to feed on eggs when NCR adults had fed on sunflower pollen. While increasing plant diversity can benefit natural enemies and pest control within agroecosystems, it is important to consider how floral resources alter dietary preferences of biocontrol agents.

**Abstract:**

Landscape diversification with flowering plants can benefit pollinators and natural enemies, although insect pests can also use floral resources for nutrition and chemoprotection. Corn rootworms (Coleoptera: Chrysomelidae, *Diabrotica* spp.) are major pests of corn (*Zea mays* L.), and while subterranean larvae primarily feed on corn roots, adult rootworms commonly consume floral resources from other plant species. We quantified the species, density, and sex of adult corn Diabroticite rootworm beetles on wild and cultivated sunflower, corn, and squash, quantified pollen within the bodies of adult northern corn rootworms [NCR, *D. barberi* (Smith & Lawrence)], and investigated how consumption of sunflower and corn pollen by NCR adults impacted predation of their eggs by two soil-dwelling mites with different feeding specialization. NCR were the most common Diabroticite species on sunflower inflorescences and western corn rootworm (WCR, *D. v. virgifera* LeConte) were more abundant in corn and squash blossoms. Pollen feeding by NCR adults did not impact egg predation by omnivorous *Tyrophagus* *putrescentiae* (Schrank) (Acari: Sarcoptiformes, Acaridae), but predatory *Stratiolaelaps* *scimitus* (Womersley) (Acari: Mesostigmata, Laelapidae) ate eggs less frequently and took longer to feed on eggs from NCR females that had fed on sunflower pollen. This research suggests pollen feeding by adult NCR can impact predation of their eggs. While increasing plant diversity can benefit natural enemies and pest control within agroecosystems, it is important to consider how floral resources alter dietary preferences of biocontrol agents.

## 1. Introduction

Landscape diversification can enhance agricultural production [1] and adding flowering plants within or around agricultural fields benefits pollinators and natural enemies [2,3,4,5]. However, insect pests are also found on non-crop plants and consume floral resources that can positively impact their development, longevity, and reproduction [6,7,8,9,10]. Pollen in particular is rich in protein, lipids, and vitamins [11,12] but also contains numerous secondary compounds (e.g., alkaloids, terpenoids, phenolics, and tannins) that play a role in interspecific communication with pollinators and defense against pollinivores and microbes [11,13,14,15,16,17], although only a few pollens appear to be toxic to insects, a property that has been best studied in bees [12]. Some herbivorous insects use secondary plant compounds to chemically defend themselves or their offspring [18]. Adult and immature leaf beetles (Chrysomelidae) are particularly well-known for chemoprotection and can biosynthesize defensive chemicals or sequester them or their precursors from food plants [19,20,21,22]. Within some chrysomelid taxa, both sexes contribute to chemoprotection, with females sequestering chemicals within eggs or associated secretions (e.g., mucilage) and males passing compounds to females during mating [23,24,25,26].

Northern corn rootworms [NCR, Chrysomelidae: *Diabrotica barberi* (Smith & Lawrence)] and western corn rootworms (WCR, *D. virgifera virgifera* LeConte) are significant pests of corn (*Zea mays* L.) [27,28]. Rootworm larvae eat corn roots, thus negatively impacting plant physiology and yield [27,28]. In the Midwest, rootworm adults emerge from the soil from July through September [29,30] and feed on aerial corn tissue, including pollen, silks, immature ears, and leaves [31,32,33]. As corn plants mature and become a less attractive food source, rootworms adults consume pollen sources from variety of weeds and grasses within or outside corn fields [31,32,33,34,35,36], with emigration from fields being influenced by adult density [37].

Adult *Diabrotica* have been recorded from several species of Cucurbitaceae (e.g., cucumbers, pumpkins, and squash) [6,38,39]. Cucurbit blossoms (although not pollen) contain chemicals called cucurbitacins that are kairomones and feeding stimulants for multiple Diabroticite species (i.e., rootworm beetles within the tribe Luperini) [38,40,41]. WCR sequester cucurbitacin metabolites, including in their hemolymph and eggs [42,43], which can provide protection against predators [44,45]. Males of a closely related species, *D. undecimpunctata howardi* Barber (SCR), transfer cucurbitacins to females in spermatophores, with the majority subsequently sequestered in eggs [25], and eggs and larvae containing these chemicals are less susceptible to an entomopathogenic fungi [24]. While NCR also feed on cucurbits [46], they are less prevalent than WCR [47], are less sensitive to cucurbitacins than WCR, and have different levels of attraction to various plant kairomones [41,48].

NCR are native to North America, and are associated with prairie grasslands in addition to corn [49,50]. NCR, especially females, are thought to move more readily to non-corn habitats and have a broader host range than WCR [32,51,52]. NCR adults feed on several host plants [6] but are common on Asteraceae inflorescences, especially sunflowers (*Helianthus* spp.), where they consume floral resources [31,51,53,54]. In the United States, adult WCR tend to be less common on sunflower compared to NCR, even if the former is abundant in the general area [51,54] and WCR prefer corn and squash pollen over sunflower and goldenrod pollen [55]. Sunflower pollen is lower in protein compared to other pollens, but rich in secondary metabolites, particularly alkaloids [15,56].

Adult female rootworms usually return to cornfields to oviposit and lay clutches of eggs in the soil that overwinter and hatch in the spring [33,57,58]. However, even though eggs have an extended period of vulnerability to predators and pathogens, mortality of first instar larvae is an important factor limiting rootworm populations [59]. Several microarthropods feed upon rootworm immatures, including predaceous and omnivorous mites [60,61,62,63,64,65,66]. Gould and Massey [67] posited that cucurbitacins could protect Diabroticite eggs from subterranean predators.

Chemoprotection is common within the Chrysomelidae and *Diabrotica*, but given the apparent differences in adult feeding behavior between NCR and WCR, NCR may acquire protective chemicals from different plant taxa. The first objective of this study was to quantify the species identity, sex, and abundance of adult *Diabrotica* associated with sunflowers in a field setting and determine if adults were consuming pollen by examining their gut contents. Because of previous field observations, we hypothesized that NCR would be more abundant on sunflowers than other *Diabrotica* spp., that females would be more abundant than males, and that both sexes would have sunflower pollen in their guts. Our second objective was to determine how supplementing the diet of adult NCR with sunflower or corn pollen impacted egg predation by two soil-dwelling mites with different levels of dietary specialization (generalist predator versus omnivore). We hypothesized that ingestion of either sunflower or corn pollen by NCR adults would reduce predation of their eggs by mites, regardless of mite identity.

## 2. Materials and Methods

### 2.1. Observational Field Surveys of Diabroticite Beetles

We conducted two field surveys to examine associations of adult Diabroticite beetles (Galerucinae, Luperini, Diabroticina) with sunflower. The first (i.e., seasonal survey) focused on sampling across multiple dates in one location with multiple types of host plants (sunflower, corn and cucurbits), while the second assessed Diabroticite beetles on wild sunflower over a larger area (i.e., geographic survey).

#### 2.1.1. Seasonal Survey

We determined the species identity, density, and sex ratio of adult Diabroticite beetles associated with cultivated sunflowers (*H. annuus* var. *macrocarpus*), wild sunflowers (*H. annuus* var. *annuus*), corn, and squash (*Cucurbita* spp.) throughout August 2007. The field site was located on the Eastern South Dakota Soil and Water Research Farm (ESDSWRF) near Brookings, SD. There were two adjoining blocks of hybrid sunflowers (38.1 × 12.2 m), each with 16 rows spaced 76.2 cm apart. We planted the northern-most block on 4 June 2007 with 8377 NuSun™ (Mycogen^®^ Seeds, DowAgroSciences LLC, Indianapolis, IN, USA) at 81,315 seeds/ha with 22.9 cm within row plant spacing, and planted the southern block on 15 June 2007 with a mix of 8377 and 63M80 (Pioneer Hi-Bred, Johnston, IA, USA). The cultivated sunflower was bordered on three sides by mown grass and on one side by a 36.6 × 45.7 m block of corn. This site had been continuously planted with corn since 2000. On 14 May 2007, we planted corn (DeKalb^®^ 46-26, Monsanto, St. Louis, MO, USA) at 81,315 seeds/ha with 76.2 cm between row spacing and 17.5 cm within row spacing. Wild sunflowers bordered three sides of the corn block. A block of squash (9.1 × 12.2 m) was nested within the NW corner of the corn block, comprised of five rows running N-S with 1.5 m between rows, five hills per row, and 2.1 m between hills. We hand-planted seeds on 22 May 2007 with three to four seeds per hill. There were two rows of acorn winter squash (*C. pepo* L., ‘Bush Acorn-Table King,’ Bentley Seeds, Cambridge, NY, USA), one row of buttercup winter squash (*C. maxima* Duchesne, ‘Burgess Buttercup,’ Ferry-Morse Seed Company, Fulton, KY, USA), and two rows of zucchini summer squash (*C. pepo*, ‘8-Ball’ and ‘1-Ball,’ Ferry-Morse Seed Company, Fulton, KY, USA).

We sampled cultivated sunflowers on 2 and 6 August from the early planted block and on 10 August and 15 August from the late planted block. On each date, we sampled 10 plants from each of five linear N-S transects for a total of 50 plants. We sampled wild sunflowers on 2, 6, 10 and 15 August, with one inflorescence taken from an individual plant (*n* = 39 on 2 August and *n* = 50 for the other dates). We collected all sunflowers inflorescences at the R5 or early R6 growth stage (i.e., flowering) [68] by placing a plastic bag over the inflorescence and cutting the stem. Bags were labeled and frozen (−20 °C) until we processed samples, which involved quantifying inflorescence diameter and determining Diabroticite beetle species identity, sex, and abundance. We used a ruler and measured from the top to the bottom of the sunflower disc (excluding the ray petals), then rotated the head 90° and took another measurement, which we used to calculate an average inflorescence diameter. Body morphology, including basitarsal pads, was used to identify male versus female rootworms [69]. On each sampling date, we assessed sunflower pollen feeding for 10 NCR males and 10 NCR females from both cultivated and wild sunflower samples. Adults were rinsed in 70% ethanol and tweezers and insect pins used to dissect out the gut, which was then mounted in Hoyer’s solution on a glass microscope slide under a coverslip [70]. We used a compound microscope to examine slides and consulted reference books to confirm the presence or absence of sunflower pollen [71,72].

Diabroticite beetle identity, sex, and abundance were also assessed in adjacent corn and squash plots. In the corn plot, 20 yellow sticky traps (Pherocon^®^ AM No-Bait, Trécé Inc., Adair, OK, USA) were placed every two to three rows along two linear transects extending diagonally from each corner. There was a 1.5 to 2.3 m buffer zone between outermost traps and plot edges. Wooden stakes were sunk into the ground within the row between corn plants and traps were secured to stakes approximately 1.5 m above the ground using large binder clips. Traps were collected on 2, 9, 15 and 24 August. Beetles were removed using a metal spatula and placed ventral side up on paper for identity and sex determinations. Squash plants were sampled on 10 and 17 August. All open flowers were collected, and as samples were limited and plants were intertwined, multiple flowers were taken from the same plant. On the first date, 26 flowers were collected from both acorn and butternut squash plants, while 10 and nine flowers were taken from 8- and 1-Ball, respectively. On the last date, 24 and 25 flowers were sampled from acorn and butternut squash plants, while 10 were taken from both 8- and 1-Ball varieties. Flowers were placed in separate plastic bags and transported to the lab in coolers, where we then assessed rootworm identity, sex, and density.

#### 2.1.2. Geographical Survey

**Figure 1:**
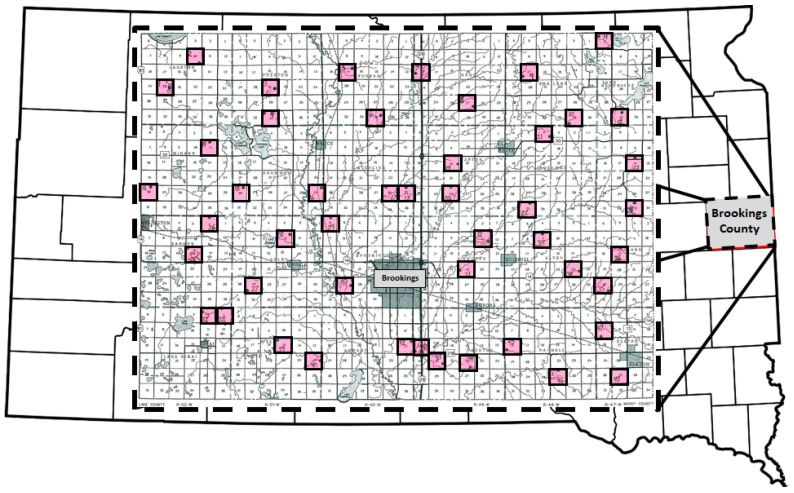
Location of sample sites in the geographic survey of Brookings County, SD. Shaded squares with bold outlines indicate the townships in which wild sunflowers were sampled.

The following year, adult Diabroticite beetles associated with wild sunflowers were surveyed at multiple sites across a broader area, specifically Brookings County, SD (Figure 1). During 25–27 August 2008, 48 sites were sampled, with two sites located in each county township. Sites were in uncultivated field margins next to corn, soybean, alfalfa, grass, wheat or a combination of the above vegetation. The majority of wild sunflowers sampled were common sunflower, (*H. annuus* var. *annuus*; 67.1%, *n* = 322 of 480), although Maximilian sunflower (*H. maximiliani* Schrad., 14.1%, *n* = 68 of 480) and a *Helianthus* species whose identity is unconfirmed, but believed to be Jerusalem artichoke (*H. tuberosus* L., 18.8%, *n* = 90 of 480) were also sampled [73,74]. Ten inflorescences were sampled at each site, with each inflorescence clipped from a different plant and placed into a separate plastic bag. Samples were transported in coolers to the lab where they were frozen at −20 °C. A dissecting microscope was used to determine adult rootworm identity, sex, and density. A subsample of male and female NCR (*n* = 229) were examined for the presence or absence of sunflower pollen, as described previously.

### 2.2. NCR Egg Predation Experiments

Lab choice experiments were used to explore impacts of pollen feeding by NCR adults on predation of their eggs by soil mites. We fed field-collected sunflower and/or corn pollen to lab-reared NCR adults and collected their eggs, which were subsequently used in predation experiments.

#### 2.2.1. Pollen Collection

Sunflower pollen was field collected on 2 August 2007 from plants used in the seasonal survey (variety 8377 NuSun™ Mycogen^®^ Seeds). Sunflower heads (R5 growth stage) were removed and placed in a greenhouse (30 ± 2 °C, 16L:8D, 40–60% RH) to dry for 1d, after which pollen was manually scraped off using a metal spatula or by gently rubbing two flower heads together. Pollen was sieved twice to remove debris (250 µm and 425 µm opening sizes) and heated in a metal pan on a plate warmer for 3 d to remove any live insects that were present (e.g., thrips). Corn pollen was collected during anthesis (VT-R1 growth stage, DeKalb^®^ 46-26). Brown paper bags were placed over corn tassels on 20–24 July, folded over the tassel base, secured with staples, and removed after three days. Pollen was then shaken through a series of sieves to remove debris (1.18 mm, 250 µm, and 425 µm opening sizes). After inspecting corn and sunflower pollens for contaminants under a dissecting scope, they were placed in separate plastic containers (15 dram clear snap cap vials) and stored in the freezer (−20 °C).

#### 2.2.2. Collection of NCR Eggs

We used NCR adults from a colony that was founded several years prior to experiments from adults field collected in Brookings county, SD that were subsequently maintained at the USDA-ARS Research Lab (Brookings, SD, USA) using rearing procedures based on WCR rearing protocols [75,76]. There were four diet treatments: (1) control, no pollen, (2) sunflower pollen added, (3) corn pollen added, and (4) mixed, sunflower and corn pollen added. Treatments were randomly assigned to experimental cages of NCR adults, with four cages per treatment. Cages consisted of a 0.31 m^3^ wooden frame covered with 18 mesh screen and were maintained for three weeks at room temperature (25 ± 2 °C, 14L:10D, 60 ± 5% RH). Pollen was added to non-control cages three times a week on the lids of 15 dram plastic snap-cap vials. Each cage that received a single pollen type (sunflower or corn) received 0.6 mL per time period, while mixed cages received 0.3 mL sunflower pollen and 0.3 mL corn pollen. All cages received two patties (~6 to 8 mL) of artificial diet, which is also used to rear WCR, and consisted of: 3640.0 g of dry diet powder (F9768B-M, Bio-Serv, Frenchtown, NJ, USA) mixed with 14.6 g lincomycin-spectinomycin antibiotic (L-S 50, Pharmacia & Upjohn Co., Kalamazoo, MI, USA), 400 mL glycerin (5500, Bio-Serv), and 5.0 L distilled water. Diets were dispensed on wax paper in small patties, allowed to dry overnight, then transferred to plastic bags and stored at 4 °C. Moisture was supplied in the form of agar bars (agar set within a 2.5 × 10 cm plastic matrix) twice a week.

Approximately 100 NCR females (*n* = 96 to 102) and 50 NCR males (*n* = 50 to 53) from a newly emerged same-age cohort were added to each cage. Each cage received one oviposition dish, which were replaced on a weekly basis. Oviposition dishes consisted of sterile plastic Petri dishes (15 × 60 mm; 351007 BD Falcon^®^, Becton Dickinson Labware, Franklin Lakes, NJ, USA), filled with 10 mL of finely sifted soil (80 mesh) topped with 10 mL of soil aggregates (≤ 6.5 mm). Prior to being added to experimental cages, 7 mL of distilled water was added to each dish using a graduated cylinder, and dishes were covered (lid with one central 8 mm hole) to let the moisture distribute evenly throughout the soil. Dishes were covered with a rectangular piece of fluted stainless steel (approximately 6.5 × 7.5 cm, 2.0 cm high) to shade the oviposition site [75]. After oviposition, dishes were removed from experimental cages, non-perforated lids were placed on dishes and labeled, dish edges were covered with Parafilm^®^ (Pechiney Plastic Packaging, Menasha, WI, USA) to prevent desiccation, and dishes were transferred to a 25 °C incubator in constant darkness for 7 to 14 d to harden the eggs.

Washing and floating methods were used to recover eggs from oviposition dishes within two weeks after removing them from cages [75,76]. Dish contents were individually washed through a 1.7 mm into a 250 µm sieve. Material caught in the latter sieve was backwashed into a plastic container using a saturated saltwater solution. Additional saltwater was added, the floating material strained through another 250 µm sieve, and then backwashed into Petri dishes (120 × 60 mm) filled with sifted 80 mesh soil and placed in a refrigerator (0L:24D, 4 °C) until used in predation experiments. Eggs were then washed out of the soil and kept in a refrigerator (0L:24D, 4 °C) on moist filter paper within separate Parafilm covered Petri dishes (120 × 60 mm) nested within sealed plastic containers.

#### 2.2.3. Confirmation of NCR Pollen Feeding

Two methods were used to confirm that lab-reared NCR adults consumed offered pollen. For experimental cages, this was confirmed qualitatively by visually observing beetle feeding behavior, pollen disappearance, and color of beetle frass (bright yellow or orange). We also established separate cages in the same location using a similar experimental design to quantify pollen consumption by captive adult NCR. Cages were plastic containers (16 oz) with fine mesh stretched across the top secured with a rubber band. Five male and five female lab-reared NCR from a newly-emerged same age cohort were added to each cage. There were eight replicates of three diet treatments, sunflower pollen only (0.6 mL), corn pollen only (0.6 mL), and a combination treatment with both sunflower and corn pollen (0.3 mL of each). Pollen was offered in the detached lid of a 1.5 mL microcentrifuge tube and was replaced once a week. Twice a week cages were provisioned with one artificial diet patty and agar for moisture. Cages were maintained at 25 ± 2 °C, 14L:10D, 60 ± 5% RH. As beetles died, they were removed from cages and placed in 4 dram glass vials with 70% ethanol. The experiment was terminated after three weeks, at which time all remaining live adults were collected and preserved in alcohol.

Pollen within bodies of experimental beetles (sunflower pollen only, *n* = 75; corn pollen only, *n* = 69; both sunflower and corn pollen *n* = 69) was recovered using acetolysis, which involved dissolving the beetle’s tissues while preserving the pollen grains, followed by staining the pollen with safranin, and mounting samples on microscope slides in Hoyer’s medium [70,72,77]. Pollen grains were identified using keys and reference books [71,72] and corn and sunflower pollen within each beetle quantified using a compound microscope by counting all mounted pollen grains (i.e., all pollen grains within the beetle’s body). Of the 213 beetles used to assess pollen consumption, sex data was missing for 42 individuals.

#### 2.2.4. Predation Experiments

We purchased the predatory mite *Stratiolaelaps scimitus* (Womersley) (Acari: Mesostigmata, Laelapidae), which has also been referred to as *S. miles* or *Hypoaspis miles* [78], from Biocontrol Network (Brentwood, TN, USA). We collected the omnivorous mite *Tyrophagus putrescentiae* (Schrank) (Acari: Sarcoptiformes, Acaridae) from shipments of commercially available predatory mites where they were presumably used as a food source. Soil mite species used in choice experiments were selected because they are known to feed on rootworms and have different degrees of feeding specializations, with *S. scimitus* being a generalist predator [65,79] and *T. putrescentiae* being an omnivore that feeds on a variety of food, e.g., plants, stored products, fungi, nematodes, and southern corn rootworm eggs [60,80,81]. Mites were maintained as described in Prischmann et al. [65] in 540 mL plastic containers (Fabri-Kal Corporation, Kalamazoo, MI, USA) with a plaster base (approx. 2.5 cm) that was periodically moistened to maintain humidity. Mites were fed a mixed-prey diet of springtails (Collembola), nematodes, acarid mites, and active dry yeast (Red Star, Lesaffre Yeast Corporation, Milwaukee, WI, USA). Representative mite specimens were mounted in Hoyer’s medium on glass slides and their identity confirmed using a compound microscope and relevant keys [78,80,82,83].

Experimental arenas were designed to provide adequate humidity and ensure the enclosed mite would have a high probability of encountering all NCR eggs. Arenas consisted of 2.0 mL plastic microtubes with a 0.8 mL plaster base moistened with 400 μL of distilled water. One healthy (i.e., turgid, cream-colored) NCR egg from each diet treatment (i.e., control no pollen, sunflower pollen only, corn pollen only, sunflower and corn pollen) was placed near the side of the tube so that the four eggs were equidistant from the center and from each other. Different paintbrushes were used to move NCR eggs from each diet treatment. One mite (adult female *S. scimitus* or *T. putrescentiae*) was then added to the center of the arena using a paintbrush. Thirty control tubes with NCR eggs but without mites were used to compare the appearance of intact and diseased eggs to those that had been fed upon by mites. Microtubes were randomly arranged in plastic racks and maintained in an incubator (constant darkness, 25 ± 2 °C, 40–60% RH), and a uniform amount of distilled water was added to all arenas as needed (5 to 10 μL). Microtubes were opened and the status of the mite and rootworm eggs assessed every 24 h until the mite fed on a rootworm egg or for a maximum of 7 d. Eggs were considered fed upon if the chorion was punctured and egg deflated or if only the egg chorion remained. Any NCR eggs that may have been diseased (e.g., discolored or with whitish fuzz) were replaced daily. Mite eggs laid during the course of the experiment were removed using a fine paintbrush.

### 2.3. Statistical Analyses

We used SYSTAT version 12.02 [84] and JMP^®^13 [85] to analyze the data, with an alpha level of α = 0.05. We used graphical exploration, including histograms, and Levene’s test for equality of variances to assess data normality.

#### 2.3.1. Seasonal Survey

Data from each plant taxa were analyzed separately, and each inflorescence (sunflower), flower (squash), or sticky trap (corn) was considered a replicate. Beetle density data were summed across the season and converted to number of adult beetles per sampling unit (trap, inflorescence, or flower) per sample period. Data from different squash varieties were combined for analysis. Due to non-normal distributions and unequal variance among Diabroticite beetle taxa, non-parametric Kruskal-Wallis tests followed by post-hoc Steel-Dwass tests, i.e., a non-parametric equivalent to Tukey’s Honest Significant Difference test [86,87] were used to assess differences in beetle species, with density per sampling unit per sample period as the dependent variable. For determining differences in densities of male and female NCR and WCR, data were log (X + 1) transformed prior to analysis using two-tailed t-tests. For less abundant Diabroticites, [e.g., SCR, southern corn rootworm, *D. undecimpunctata howardi*: CB: striped cucumber beetle, *Acalymma vittatum* (F.)], sex data were analyzed using the non-parametric Wilcoxon test. Linear regression was used to determine if sunflower inflorescence diameter impacted the density of male and female NCR, as well as relationships between densities of female and male NCR (log X + 1 transformed).

#### 2.3.2. Geographical Survey

We used non-parametric Kruskal-Wallis and post-hoc Steel-Dwass tests to investigate differences in adult Diabroticite species identity on wild sunflowers, with density per inflorescence as the dependent variable. Each site was considered a replicate and beetle densities within each taxa were averaged among the ten plants (i.e., inflorescences) sampled per site. We then determined if male or female NCR were more abundant on wild sunflowers, using density per inflorescence (log X + 1 transformed) as the dependent variable.

#### 2.3.3. Confirmation of NCR Pollen Feeding

Due to non-normal data distributions, we used non-parametric Kruskal-Wallis tests followed by post-hoc Steel-Dwass tests to examine effects of diet treatment on the number of corn and sunflower pollen grains recovered from individual female and male NCR beetles.

#### 2.3.4. Predation Experiments

Data from each mite group (*S. scimitus* or *T. putrescentiae*) were analyzed separately, and each arena was considered a replicate. We used data from 76.0% of arenas with *S. scimitus* (*n* = 76 of 100) and 78.5% of arenas with *T. putrescentiae* (*n* = 51 of 65) in analyses. Data from 9.0% (*n* = 9 of 100) of *S. scimitus* arenas and 15.4% (*n* = 10 of 65) of *T. putrescentiae* arenas were excluded because the mite ate more than one NCR egg in a 24 h period, and 14.0% (*n* = 14 of 100) of *S. scimitus* and 6.1% (*n* = 4 of 65) of *T. putrescentiae* arenas were excluded because mites died prior to eating an egg or did not eat any eggs during the experiment. The frequency with which mites preyed upon rootworm eggs from NCR adults fed different diets was analyzed using frequency tables and Pearson’s chi-square statistic. The main effects of each independent variable (sunflower or corn pollen) on the frequency of egg consumption were assessed separately using one-way tables, and interactions between variables were assessed using two-way tables. Data on how treatments affected days until an egg was consumed were square-root transformed to normalize the data distribution. Data were analyzed using factorial ANOVA, with rootworm adult diet (presence/absence of sunflower and corn pollen) as the independent variables and number of days until an egg was eaten as the dependent variable, with Tukey’s HSD test used for mean separation.

## 3. Results

### 3.1. Seasonal Survey

Three Diabroticite beetle species were collected from corn, squash, and wild or cultivated sunflower: NCR, northern corn rootworm; *Diabrotica barberi*; WCR, western corn rootworm, *D. v. virgifera*; SCR, southern corn rootworm, *D. undecimpunctata howardi*, which is also called the spotted cucumber beetle. The striped cucumber beetle [(CB, Chrysomelidae: *A. vittatum*] was also collected from corn and squash.

**Figure 2:**
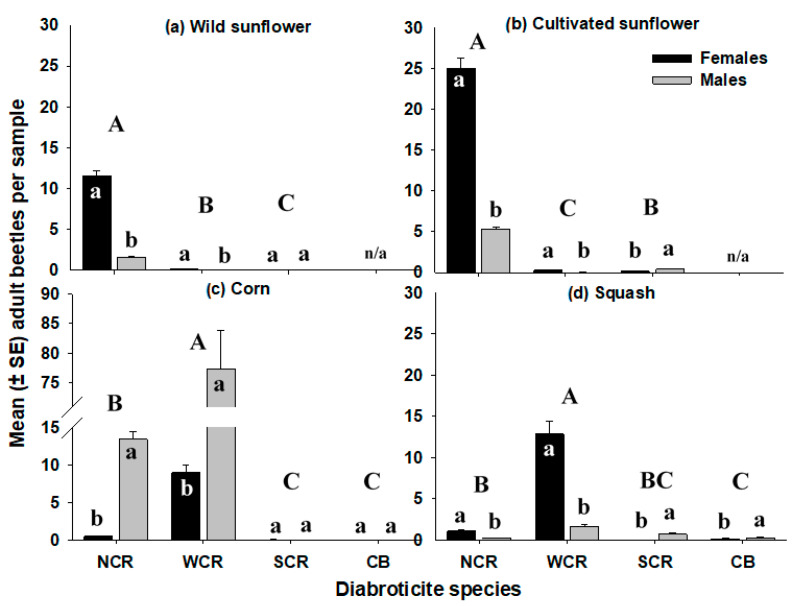
Mean densities (± SEM) of adult female and male Diabroticite beetles per sample collected in the seasonal survey from: (**a**) wild sunflower, (**b**) cultivated sunflower, (**c**) corn, and (**d**) squash. NCR = northern corn rootworm, *D. barberi*; WCR = western corn rootworm, *D. v. virgifera*; SCR = southern corn rootworm, D. *undecimpunctata howardi*; CB = cucumber beetle, *A. vittatum*. Means with the same letters are not significantly different (α = 0.05), with upper-case letters indicating differences among species and lower-case letters representing differences between females and males.

On wild sunflower, NCR was the dominant species (Figure 2a; H = 116.06, df = 2, *p* < 0.0001) and more NCR females were collected per inflorescence than males (t = −16.85, df = 55.5, *p* < 0.0001). The number of female NCR per inflorescence ranged from 0 to 48 and was consistently high throughout the season, with 8.6 ± 1.3 (mean ± SE) on 2 August, 12.1 ± 1.5 on 6 August, 12.9 ± 1.1 on 10 August, and 11.9 ± 1.3 on 15 August. The number of male NCR per inflorescence ranged from 0 to 11 and peaked at 2.7 ± 0.3 (mean ± SE) on 10 August. Densities of WCR on wild sunflower were consistently low throughout the season, with three or fewer beetles found per inflorescence and an average of 0.1 ± 0.04 (males plus females) on 10 August. Even so, there were more WCR females per inflorescence than males (t = −2.53, df = 65.1, *p* = 0.014). No CB were collected from wild sunflower, and densities of SCR were extremely low, with similar numbers of males and females (four females and two males, Z = 0.88, *p* = 0.379).

The average diameter of a wild sunflower inflorescence was 3.32 ± 0.04 cm (mean ± SE). Inflorescence diameter was not related to the density of male NCR (data not shown; adjusted R^2^ < 0.001, t = −1.04, *p* = 0.298), whereas it positively impacted densities of female NCR (adjusted R^2^ = 0.03, t = 2.76, *p* = 0.006), an effect that was strongest at the end of the season (15 August: adjusted R^2^ = 0.23, t = 3.92, *p* < 0.001), with an average 3.3-fold increase in NCR females from the smallest to largest heads. Density of female NCR was positively related to male NCR density (data not shown; adjusted R^2^ = 0.25, t = 7.91, *p* < 0.0001).

The species found on cultivated sunflower were similar to that on wild sunflower, with NCR being much more abundant than WCR and SCR (Figure 2b; H = 104.82, df = 2, *p* < 0.0001). The number of NCR females ranged from 0 to 101 per inflorescence and increased steadily over the season, peaking on 15 August at 43.8 ± 2.5 (mean ± SE). More NCR females were collected per inflorescence than males (t = 14.81, df = 52.6, *p* < 0.0001). The number of NCR males ranged from 0 to 25 per inflorescence and peaked on 10 August at 10.8 ± 0.8 (mean ± SE). Densities of WCR and SCR were low throughout the season on cultivated sunflower (ranged from 0 to 4 per inflorescence), with more WCR females collected than males (Z = −4.61, *p* < 0.0001) although for SCR males were more abundant than females (Z = 4.80, *p* < 0.0001).

The average diameter of a cultivated sunflower inflorescence was 10.46 ± 0.11 cm (mean ± SE). Density of male NCR on cultivated sunflower decreased slightly as inflorescence diameter increased (data not shown; adjusted R^2^ = 0.02, t = −2.20, *p* = 0.029), from an average of seven beetles on smaller heads to three on larger heads. The opposite pattern occurred for female NCR (adjusted R^2^ = 0.11, t = 4.98, *p* < 0.001), with the latter relationship being the strongest at the start of the season (2 August: adjusted R^2^ = 0.20, t = 3.53, *p* = 0.001) and an average 3.5-fold increase in NCR females from the smallest to largest heads, although we did not quantify the number of florets in bloom at the time of sampling for either type of sunflower. As with wild sunflower, the density of female NCR had a strong positive effect on male NCR density (data not shown; adjusted R^2^ = 0.17, t = 6.41, *p* < 0.0001). Sunflower pollen was present in the guts of all the NCR beetles dissected from wild and cultivated sunflower (*n* = 160), regardless of sampling date or sex. In contrast to sunflower, WCR were the most abundant species collected from sticky traps placed within corn (Figure 2c; H = 71.88, df = 3, *p* < 0.001) followed by NCR. Virtually no SCR or CB were collected from corn across all sample dates, and densities of males and females were similar (SCR: four females and two males, Z = −0.85, *p* = 0.394; CB: two males and one female, Z < −0.001, *p* = 0.971). More WCR males were collected per trap than females (t = 15.22, df = 36.2, *p* < 0.0001). The number of male WCR per trap ranged from 8 to 336 and declined over the sampling period from a peak of 160.1 ± 16.2 (mean ± SE) on 2 August. The number of female WCR per trap ranged from 0 to 32 and were relatively consistent across sample dates, with a modest peak in density of 11.3 ± 1.8 (mean ± SE) on 15 August. Similar patterns were found for NCR, with more males than females (t = 22.55, df = 32.7, *p* < 0.0001). The number of male NCR per trap ranged from 0 to 52 and peaked at 25.8 ± 2.9 (mean ± SE) on 2 August before gradually declining. The number of female NCR per trap ranged from 0 to 5 and remained low throughout the season. However, it is important to note that the collection method in corn (i.e., sticky traps) was different than that in sunflower and squash, and may have influenced the relative abundance of Diabroticite adults collected.

WCR was the primary species collected from squash (Figure 2d; H = 140.53, df = 3, *p* < 0.0001) with a peak density (mean ± SE) of 10.8 ± 1.6 per flower on 17 August and more WCR females collected per blossom than males (t = −9.76, df = 119.3, *p* < 0.0001). The number of female WCR per flower ranged from 0 to 106, while males ranged from 0 to 29. NCR were more abundant than SCR (*p* = 0.012) and CB (*p* = 0.002), and more NCR females were collected than males (Z = −5.51, *p* < 0.0001). In contrast, densities of SCR and CB were similar (*p* = 0.997) and more males were collected than females (SCR: Z = 7.60, *p* < 0.0001; CB: Z = 2.72, *p* = 0.007).

### 3.2. Geographical Survey

Three rootworm species were collected from wild sunflowers in Brookings county: NCR, WCR, and *Diabrotica cristata* (Harris), with the latter (10 males and one female) only found at one site in Richland township. Similarly, WCR (one male and no females) were only found at one site in Elkton township, even though corn, the larval host for WCR and NCR, bordered 48% of the sites. In contrast, NCR were found at 98% of sites. Density of rootworm species per wild sunflower inflorescence at each site varied significantly (H = 127.20, df = 2, *p* < 0.001), with higher mean (± SE) densities of NCR per inflorescence (6.19 ± 0.46) compared to *D. cristata* (0.02 ± 0.01) or WCR (0.002 ± 0.002), although densities of the latter two species were not significantly different from each other (*p* = 0.999). Densities of female NCR per wild sunflower inflorescence (5.98 ± 0.45) were higher than male NCR (0.21 ± 0.03) [F = 124.91, df = (1,51.5), *p* < 0.001]. Sunflower pollen was present in the guts of 78.6% of adult NCR beetles dissected from wild sunflower (*n* = 189 of 229; *n* = 4 of 5 males, *n* = 185 of 224 females).

### 3.3. Confirmation of NCR Pollen Feeding

The number of pollen grains recovered from a single lab-reared NCR adult ranged from 0 to 4708, and averaged 377.13 ± 44.30 (mean ± SE). Fewer than 10 pollen grains were found in a small proportion of beetles (15.1%, *n* = 28 of 185). When these individuals were temporarily excluded from the analysis, the average number of pollen grains recovered per beetle was 433.75 ± 49.70 (mean ± SE). The total number of pollen grains recovered from a single beetle varied among treatments (Pearson’s χ^2^ = 23.15, df = 2, *p* < 0.0001). Similar numbers of pollen grains were recovered from beetles in the combination (341.19 ± 47.00) and the sunflower pollen only treatments (604.00 ± 109.74; *p* = 0.992), while the amount of pollen recovered from the corn only treatment (153.48 ± 29.37) was lower than the others (sunflower only: *p* = 0.0002; combo: *p* < 0.0001). Pollen recovered was related to pollen availability, regardless of pollen identity or sex of beetle (Table 1). Similar amounts of sunflower pollen were recovered when beetles were offered sunflower pollen, either alone or in combination with corn pollen, with virtually no sunflower pollen was recovered when beetles were only offered corn pollen (all beetles: Pearson’s χ^2^ = 126.77, df = 2, *p* < 0.0001; females: Pearson’s χ^2^ = 54.04, df = 2, *p* < 0.0001; males: Pearson’s χ^2^ = 48.46, df = 2, *p* < 0.0001). Likewise, similar amounts of corn pollen were recovered from beetles offered corn pollen, alone or in combination with sunflower pollen, and virtually no corn pollen was recovered when beetles were only offered sunflower pollen (all beetles: Pearson’s χ^2^ = 132.09, df = 2, *p* < 0.0001; females: Pearson’s χ^2^ = 52.17, df = 2, *p* < 0.0001; males: Pearson’s χ^2^ = 54.89, df = 2, *p* < 0.0001). We believe the low numbers of pollen grains recovered from beetles that did not match the pollen offered (i.e., sunflower pollen in beetles only offered corn pollen and vice versa) was potentially due to air currents in the rearing room blowing around trace amounts of pollen. In the combination treatment, the percentage of total pollen grain that were sunflower was 65.22% ± 3.80 on average, although it ranged from 2–25% (*n* = 12), 26–50% (*n* = 9), 51–75% (*n* = 14), and 76–100% (*n* = 33). A corn pollen grain averages 80 μm in diameter and 1 mg of corn pollen has approximately 2000 grains [88]. Sunflower pollen is typically 25.5 μm in diameter and 1 mg of sunflower pollen has approximately 5500 grains [89].

### 3.4. Predation Experiments

**Figure 3:**
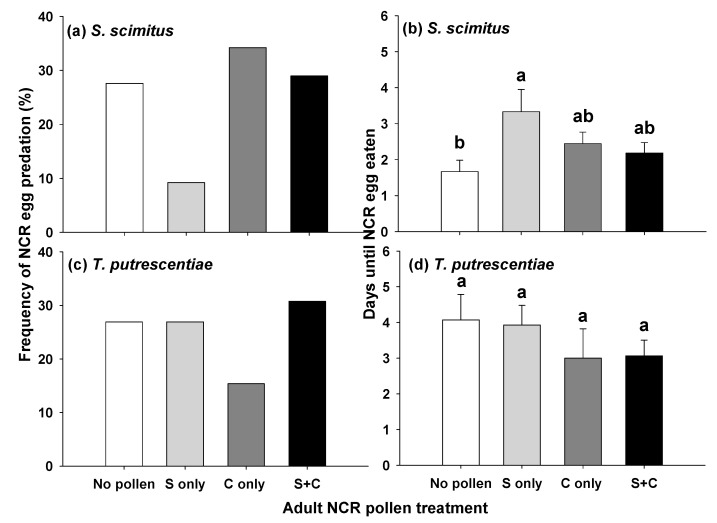
Impact of adult NCR pollen feeding on frequency of NCR egg predation for (**a**) *S. scimitus* and (**c**) *T. putrescentiae*; and days until an NCR egg was eaten by (**b**) *S. scimitus* and (**d**) *T. putrescentiae*. S = sunflower pollen only, C = corn pollen only, S + C = sunflower + corn pollen. Means with the same letters are not significantly different (α = 0.05).

In choice tests, 34.2% of the time (*n* = 26), the first type of NCR egg that the predatory mite *S. scimitus* consumed was from the corn pollen only treatment, followed by the combination (29.0%, *n* = 22), control (27.6%, *n* = 21), and sunflower pollen only treatments (9.2%, *n* = 7; Figure 3a). *Stratiolaelaps scimitus* consumed eggs more frequently when they were from NCR adults fed corn pollen (χ^2^ = 5.26, *p* = 0.022), and less frequently when NCR adults had been offered sunflower pollen (χ^2^ = 4.26, *p* = 0.039). There was a marginal interaction between the two independent variables (χ^2^ = 3.25, *p* = 0.071), indicating a tendency for corn pollen to offset negative effects of sunflower pollen on frequency of consumption of eggs from the combination treatment. The number of days (mean ± SE) until *S. scimitus* preyed upon an egg was fewest when the egg chosen was from the control treatment (1.67 ± 0.32), followed by the combination (2.18 ± 0.29), corn pollen only (2.44 ± 0.32), and sunflower pollen only treatments (3.33 ± 0.62; Figure 3b), with the latter being significantly higher than the control (*p* < 0.05). There was a significant interaction between the independent variables [Sunflower × Corn, df = (1,72), *p* = 0.029], which was driven by eggs in the combination treatment being consumed faster than eggs in the sunflower only treatment. Main effects of pollen treatment were not significant [Sunflower, df = (1,72), *p* = 0.130; Corn, df = (1,72), *p* = 0.946].

In choice tests, 30.8% of the time (*n* = 16), the first type of NCR egg that *T. putrescentiae* consumed was from the combination treatment, followed by the control (26.9%, *n* = 14), sunflower pollen only (26.9%, *n* = 14), and corn pollen only treatments (15.4%, *n* = 8; Figure 3c). There were no significant effects of pollen treatment on frequency of egg consumption by *T. putrescentiae* (Sunflower × Corn, χ^2^ = 1.47, *p* = 0.225; Sunflower, χ^2^ = 1.23, *p* = 0.267; Corn, χ^2^ = 0.31, *p* = 0.579). Pollen treatment did not significantly affect how long it took *T. putrescentiae* to prey upon an egg [Sunflower × Corn, df = (1,48), *p* = 0.921; Sunflower, df = (1,48), *p* = 0.908; Corn, df = (1,48), *p* = 0.143], and the number of days (mean ± SE) until an egg was consumed was similar among treatments: corn pollen only (3.00 ± 0.82), combination (3.06 ± 0.44), sunflower pollen only (3.93 ± 0.55), and control (4.07 ± 0.72; Figure 3d).

## 4. Discussion

Non-crop plants within agricultural areas can potentially benefit insect pests in addition to pollinators and natural enemies. While it is well known that pestiferous rootworm beetles consume floral resources from non-crop plants as adults, it is less clear how feeding on these plants might impact survival of rootworm immatures. We surveyed Diabroticite adults on sunflower, corn, and cucurbits within a vegetationally-diverse research farm as well as wild sunflower from a larger geographic area, and investigated how pollen feeding impacted predation of NCR eggs by soil-dwelling mites. We collected five Diabroticite species: NCR, WCR, SCR, CB, and *D. cristata*, with the first two species being the most abundant. We rarely found *D. cristata* adults, with a total of 11 adults collected on wild sunflower at one location bordering a crop field in Brookings county SD. This species is believed to be primarily associated with prairie habitats, and adults been collected from a wide variety of grasses and forbs, as well as corn, cucurbits, and sunflowers [6,36,90,91]. Thus, our low densities may be reflective of the habitat we sampled. However, Wiesenborn and Krysan [91] found *D. cristata* adults absent or sparse at three relic prairies in Brookings county, and at the one site where they were considered abundant there was an average of 17 to 23 adults per 50-sweep transect.

Our study adds to the literature supporting the use of sunflower by adult NCR and weaker association of WCR with this plant. Densities of female NCR on sunflower were higher than males, but this could be influenced by sampling time, as males emerge first and the sex ratio is female biased later in the season [57,92]. In Europe, adult WCR feed on sunflower [35,93] and it has been suggested their host plant range is broader than in the U.S. [35]. Although WCR are considered a sunflower pest in some locations in Europe [94], severe widespread damage has not been reported, even in corn [95]. In the midwestern U.S., WCR appears to have a greater association with corn [32,49,96], although Campbell and Meinke [36] observed both NCR and WCR on *Helianthus*. In our studies WCR was the dominant Diabroticite species in corn, although it was largely absent from sunflower, which parallels results from Bredeson and Lundgren in South Dakota [97], Hill and Mayo [51] in Nebraska, and McKone et al. [54] who surveyed *Diabrotica* on prairie remnants near cornfields in Minnesota. For the latter study, the average NCR density was approximately 12 adults per wild type *H. annuus* head on potted plants placed at the edge of corn fields. Likewise, Dickinson and McKone [53] identified insects associated with tall-grass prairie flowers, and found high densities of NCR on *H. rigidus* (currently known as *H. pauciflorus*), the stiff or prairie sunflower. Other researchers have also noted adult NCR on *Helianthus* inflorescences [31,36,98], including a high proportion of females [51,99]. Note that *D. barberi* was elevated to the rank of species in 1983 [100], and thus some of the older records on host plant associations refer to NCR as *D. longicornis* (Say), or the subspecies *D. longicornis barberi* Smith and Lawrence [6,100,101].

We recovered sunflower pollen from adult female and male NCR field-collected from sunflowers, and lab-reared NCR adults readily consumed both sunflower and corn pollen. Cinereski and Chiang [31] determined corn pollen was retained in the NCR digestive tract for at least three days after pollen-feeding in a lab experiment; thus, it is likely sampled beetles had recently consumed sunflower pollen. NCR adults were observed feeding on sunflower pollen, anthers, nectar, ray petals [53,54], and leaves in the field [102], and pollen from Asteraceae has been recovered from the digestive tract of field-collected adult female and male NCR [31,32]. McKone et al. [54] observed that NCR focuses on pollen feeding, especially in the morning, and found evidence that this reduced *Helianthus* seed set. Although we did not assess pollen feeding by WCR adults, they have been observed feeding on *Helianthus* pollen and petals in the field, and sunflower pollen has been recovered from their guts [35]. Some of the compounds in sunflower pollen (e.g., flavonols, lipids, and polyamides) are feeding stimulants for WCR [103], along with primary metabolites (i.e., amino acids), although WCR prefer corn and squash pollen over sunflower and goldenrod pollen [55], and sunflower inflorescences possess chemicals that are neurotoxic or antifeedants for WCR [104].

WCR was the dominant rootworm species associated with cucurbit blossoms at our field site. Adult WCR, SCR, and other Diabroticite species sequester cucurbitacins or their precursors in their tissues, hemolymph, spermatophores, and eggs [24,25,43,44,45], which can protect adults and immatures against predators [44,45], parasitoids [105], and entomopathogens [24], although see Gould and Massey [67] and Brust and Barbercheck [106]. Robert et al. [22] demonstrated WCR also use corn root-derived plant toxins (i.e., benzoxazinoid glucosides) to protect themselves against nematodes. WCR, SCR, and other chrysomelid larvae have sticky, repellent hemolymph that protects them from predators [20,107], particularly chewing predators [108,109].

In our lab experiments, NCR pollen feeding affected egg predation by the polyphagous soil-dwelling predatory mite, *S. scimitus*, but not the more omnivorous *T. putrescentiae*. The latter is known to feed on SCR eggs [60] and laelapid mites, including in the genus *Stratiolaelaps*, attack NCR and WCR eggs [63,64], with *S. scimitus* consuming WCR eggs under lab conditions [64]. However, Pasquier et al. [110] did not find that *S. scimitus* attacked WCR eggs in lab experiments. *Tyrophagus putrescentiae* feeds on a wide variety of food, e.g., plants, stored products, fungi, and nematodes [80,81], and is able to adapt and persist on nutritionally-different diets [111] and tolerate some mycotoxins and plant-based spices [112], which could be why NCR egg consumption was similar among treatments for this mite species. In contrast, when adult NCR rootworms fed on sunflower pollen, *S. scimitus* consumed their eggs less frequently and took longer to feed on eggs, a finding that could be strengthened by conducting no-choice assays. One possibility is that NCR females were obtaining protective chemicals from the sunflower pollen that were incorporated within eggs or secretions covering eggs. We washed eggs used in predation experiments with water and handled them with paintbrushes, which could have removed materials from the egg surface, and so our results may be conservative. Chemically protecting eggs has been documented for multiple insects [26], and egg chemoprotection is common in chrysomelid beetles, with defense compounds used extending beyond cucurbitacins [20,23]. Although pollen contains several secondary compounds [11,13,14,16,17], and sunflower pollen is rich in alkaloids [15,56], there are few studies indicating pollen is the source of chemoprotective chemicals for insects. *Heliconius* butterflies, which are unusual in that they consume pollen (including from plants within the Cucurbitaceae), contain cyanogenic glycosides and are more unpalatable than closely-related non-pollen feeding species [113,114], which is attributed to the pollen providing amino acids needed to biosynthesize the defensive compounds [115,116]. There is also the possibility that the field-collected pollen we fed to NCR adults was contaminated with pesticides, atmospheric pollutants [14,117], or other plant compounds, e.g., sesquiterpene lactones and diterpenes from sunflower glandular trichomes [118,119].

Providing NCR adults with corn pollen in addition to sunflower pollen appeared to make their eggs more attractive to *S. scimitus* than sunflower pollen alone. We recovered similar densities of pollen from beetles in the two groups, and although the mean number of sunflower pollen grains recovered from NCR adults in the combination treatment was lower than when sunflower pollen was offered alone, the difference was not statistically significant. Although we only sampled individual beetles at one point in time, some beetles offered both pollens had a higher percentage of corn pollen compared to sunflower pollen (or vice versa), which could have influenced the results.

Pollen-feeding improves the survival and fitness of multiple arthropod species [9,12], and consuming corn pollen can benefit NCR and WCR survival and egg production [120,121,122]. Sunflower heads with ray florets, and squash blossoms had similar effects on NCR longevity compared to diets of sweet corn ears [52] and NCR longevity and fecundity was not significantly different on diets of corn tissue (silks, young ear tips or tassels), sunflower inflorescences, squash blossoms, or WCR artificial diet [123]. In our lab experiment, consumption of pollen by NCR adults may have differentially affected the nutritional quality of their eggs and potentially influenced predation by *S. scimitus* in a manner unrelated to chemical defense.

Understanding adult rootworm feeding behavior and nutritional ecology is important because nutritional status can impact adult fitness [52,120,123,124], root damage by the next generation of larvae [125], and invasions into new areas [122]. It is also important to consider how the presence of alternative host plants impacts pest management by influencing interactions between herbivores and their natural enemies. Our research suggests that pollen-feeding by NCR adults can reduce predation of their eggs by some soil mite species, which could disrupt biological control. Since NCR adults feed on a wide variety of weeds and non-crop plants, the impact of adult diet on the fate of rootworm immatures likely extends beyond sunflower pollen and what is known from the literature. The use of alternate host plants by herbivorous pest insects for chemoprotection deserves further study, especially as flowering plants are common in agricultural landscapes.

## Figures and Tables

**Table 1:** Amount of sunflower and corn pollen recovered from lab-reared NCR adults.

Pollen Offered	Number of Pollen Grains Recovered per NCR Beetle (Mean ± SE)					
	All Beetles (*n* = 213)		Female Only (*n* = 88)		Male Only (*n* = 83)	
	S	C	S	C	S	C
S only	603.9 ± 109.8b	0.1 ± 0.1a	414.0 ± 161.6b	0.1 ± 0.03a	717.3 ± 184.1b	0.2 ± 0.1a
C only	1.2 ± 0.3a	152.3 ± 29.4b	1.1 ± 0.3a	119.1 ± 41.7b	1.5 ± 0.7a	139.0 ± 48.8b
S + C	248.1 ± 40.5b	106.1 ± 19.2b	235.4 ± 65.9b	143.2 ± 28.3b	179.7 ± 42.3b	63.3 ± 32.5b

S = sunflower, C = corn. Within a column, means followed by different letters are significantly different at α = 0.05.

## Data Availability

The data presented in this study are available on request from the corresponding author.
